# Role of the employment status and education of mothers in the prevalence of intestinal parasitic infections in Mexican rural schoolchildren

**DOI:** 10.1186/1471-2458-6-225

**Published:** 2006-09-06

**Authors:** Luis Quihui, Mauro E Valencia, David WT Crompton, Stephen Phillips, Paul Hagan, Gloria Morales, Silvia P Díaz-Camacho

**Affiliations:** 1 Department of Human Nutrition. Centro de Investigación en Alimentación y Desarrollo, A.C. Hermosillo, Sonora, México; 2 Institute of Biomedical and Life Sciences. Division of Infection and Immunity. University of Glasgow. Scotland, UK; 3 Department of Public Health. Universidad Autonoma de Sinaloa. Culiacán Sinaloa, México

## Abstract

**Background:**

Intestinal parasitic infections are a public health problem in developing countries such as Mexico. As a result, two governmental programmes have been implemented: a) "National Deworming Campaign" and b) "Opportunities" aimed at maternal care. However, both programmes are developed separately and their impact is still unknown. We independently investigated whether a variety of socio-economic factors, including maternal education and employment levels, were associated with intestinal parasite infection in rural school children.

**Methods:**

This cross-sectional study was conducted in 12 rural communities in two Mexican states. The study sites and populations were selected on the basis of the following traits: a) presence of activities by the national administration of albendazole, b) high rates of intestinal parasitism, c) little access to medical examination, and d) a population having less than 2,500 inhabitants. A total of 507 schoolchildren (mean age 8.2 years) were recruited and 1,521 stool samples collected (3 per child). Socio-economic information was obtained by an oral questionnaire. Regression modelling was used to determine the association of socio-economic indicators and intestinal parasitism.

**Results:**

More than half of the schoolchildren showed poliparasitism (52%) and protozoan infections (65%). The prevalence of helminth infections was higher in children from Oaxaca (53%) than in those from Sinaloa (33%) (p < 0.0001). *Giardia duodenalis *and *Hymenolepis nana *showed a high prevalence in both states. *Ascaris lumbricoides*, *Trichuris trichiura *and *Entamoeba hystolitica/dispar *showed low prevalence. Children from lower-income families and with unemployed and less educated mothers showed higher risk of intestinal parasitism (odds ratio (OR) 6.0, 95% confidence interval (CI) 1.6–22.6; OR 4.5, 95% CI 2.5–8.2; OR 3.3, 95% CI 1.5–7.4 respectively). Defecation in open areas was also a high risk factor for infection (OR 2.4, 95% CI 2.0–3.0).

**Conclusion:**

Intestinal parasitism remains an important public health problem in Sinaloa (north-western Mexico) and Oaxaca (south-eastern Mexico). Lower income, defecation in open areas, employment status and a lower education level of mothers were the significant factors related to these infections. We conclude that mothers should be involved in health initiatives to control intestinal parasitism in Mexico.

## Background

Intestinal parasitic infections are still a serious public health problem in the world, particularly in developing countries [1-4]. These infections are the result of inter-related social, economic, cultural, historical, and political factors [5] Control strategies involving improved drinking water supplies, excreta disposal, sewage management, sanitation, and education have been related with reduced prevalence of intestinal parasitism [6]. Recently, concurrent programmes of nutrition, immunization, family planning, and de-worming have been shown to effectively promote health by influencing the knowledge, perceptions, and behaviour of mothers toward intestinal parasitic infections in countries like Thailand, Guatemala, Uganda, and Egypt [7-10]. These programmes are effective in areas where mothers are trained to give guidance to children and to organise volunteer groups to solve common health problems. In Mexico, a social program targeting mothers has been implemented to ensure the well-being of low-income families [11], but its impact has not been evaluated. Here, we investigated the impact of socio-economic factors and the role of the economic status and education of mothers in particular, in the transmission of intestinal parasites in rural schoolchildren in two different Mexican regions.

## Methods

### Study population

This was a cross-sectional study conducted from September, 1997, to October, 1998, in twelve rural communities of Sinaloa (Imala, Las Puentes, Pueblo Nuevo, El Treinta, Doroteo Arango, El Paraiso and El Higueral), and from September, 1999, to July, 2000, in five communities of Oaxaca (Lomas de San Jacinto, La Era, La Lobera, Pluma Hidalgo, and Sta. Maria de Magdalena). Sinaloa and Oaxaca are located in north-west and south-east Mexico, respectively, and are 1,741 kilometers apart from each other. Sinaloa and Oaxaca are 53 and 1,600 meters above sea level, and the mean annual temperatures and humidities are 25°C and 70% (Sinaloa) and 27.5°C and 75% (Oaxaca). The study sites and population were selected on the basis of a) albendazole administration to the schoolchildren by the national campaign of the Mexican government, b) an overall high rate of intestinal parasitism in the population, c) a lack of or irregular medical assistance, and d) the communities having a population less than 2,500 [12]. Sewage disposal system was not available in these communities.

### Recruitment

Official meetings with personnel from Health Services, city councils, and primary schools, as well as with parents and schoolchildren from the study sites, were carried out in order to explain the protocol of the study. School children 6–10 years, with no disabilities, or on antiparasitic treatment were included in this study. Initially, 970 children accepted to participate voluntarily but 267 were rejected during the study because they either gave fewer than 3 faecal samples (23%), had contaminated faecal samples (4%), or decided not to participate (20%). A total of 507 children, 70% (356) from Sinaloa and 30% (151) from Oaxaca, met the study requirements. The children in the study represented 43% of 1,185 officially enrolled children aged 6–10 years in the 12 primary schools visited. Written consent was required from both parents in order for the children to participate. Children requiring medical assistance were properly treated or referred for medical attention. Approval was obtained from the ethical committees of the institutions involved and the Public Health Ministries of Sinaloa and Oaxaca.

### Data collection

Collection of the socio-economic characteristics of the children's families was undertaken with a structured questionnaire that was previously pilot-tested in the study setting in order to detect and correct problems. The interviews were administered face-to-face with mothers in the children's schools. Training of interviewers was done by a local leader to minimize bias. Employment status and the level of education of the parents, house structural materials, sanitation facilities, type of drinking water (tap, commercial chlorinated or boiled water), and family income were collected as proxy variables of socio-economic status. The children's ages were obtained through birth certificates.

### Measurement of socio-economic data

Socio-economic information included questions related to civil status of the parents (unmarried, married, divorced and widow), current occupation (housewife, unemployed and skilled/unskilled job), level of school education (primary, secondary, technical training, university and others) household conditions (construction materials for wall, floor and roof, number of rooms, family size), sewage system in the house (flushing toilet, latrine, discriminate defecation), drinking water (untreated piped water, chlorinated/boiled water) and family income (amount and sources). The dependent variable was the absence (0) or presence (1) of intestinal parasite infection and (0) or (1) when intensity of helminth infection was < 200 epg and >200 epg respectively. The independent variables were age, gender, socio-economic status assessed by employed (0) or unemployed (1) parents; education of parents in years assessed on a scale ranging from 1 to 6 years of primary school, incomplete primary (0), complete primary (1). Household conditions were assessed as the type of material used for walls, roofs, and floors. Sanitation facilities were assessed as defecation in open area (0), pit/latrine (1); drinking water was assessed as tap water (0) or treated water (chlorine/boiled) (1). Family size and crowding index (number of people per room) were categorized into smaller than 5 people (0) and larger than 5 people (1). Family income was assessed as a continuous variable and estimated in number of minimum daily-wages by dividing the daily family income by the minimum daily wage.

### Collection and processing of stool samples

A total of 1,521 stool samples were collected from the children (three per child from different days). Clean plastic containers with cap and labels were used. Stool samples contaminated with water or urine were rejected. Iceboxes were used to store the stools during sampling and transportation. Each stool sample was analysed by the methods of Faust and Kato Katz according to international recommendations [13,14]. The intensity of infection was estimated indirectly by counting the number of eggs per gram of faeces (epg).

### Analysis

Data were analyzed using NCSS 2000 software (NCSS Statistical Software, Kaysville, UT). Descriptive statistics were expressed as mean and standard deviation (SD) in the case of age, years of primary education, crowding index and family monthly income. Proportions were used for prevalence, gender, employed parents, type of house material and sanitation facilities. The significance of differences in frequency distributions was tested by chi-square analysis. Means for the ages of the schoolchildren, years of primary education of the parents and crowding index in Sinaloa and Oaxaca were assessed by the student t-tests. Epg were expressed in arithmetic means with their confidence intervals and analysis of variance (ANCOVA) was used to compare the log transformations of the eggs counts by state controlling for age and gender of the children. The association between predictor variables (independent variables) with infection (dependent variable) was determined by odds ratios and their 95% confidence intervals. Stepwise method of multiple regression analysis was used to find subsets of statistically significant predictors of both intestinal parasitic infection and intensity of helminth infection. Employment status and education level of the parents, number of individuals per family, crowding index, type of materials used for walls, floors, and roofs, type of drinking water, practice of defection in open areas, and family income were entered simultaneously in backward elimination, controlling for age and gender of the children. All these factors were expected to influence the probability of infection [15]. Association of parasite infection and intensity of helminth infection with socio-economic variables were assessed by multinomial logistic regression.

## Results

The schoolchildren had an average age of 8.2 (1.4) years. In all, 289 (57%) schoolchildren were infected with two or more intestinal parasites (Table 1). Two hundred and sixty nine (53%) and 319 (63%) children were infected with protozoan (63%) and helminth (53%) intestinal parasites respectively. The pathogenic *Giardia duodenalis *(*G duodenalis*) and *Hymenlepis nana *(*H. nana*) [24% (122) and 23% (117) respectively] and the non-pathogenic *Entamoeba coli *(*E. coli*) and *Endolimax nana *(*E. nana*) [46% (233) and 38% (193)] were the most prevalent. *Ascaris lumbricoides *(*A. lumbricoides*), *Trichuris trichiura *(*T. trichiura*) and *Entamoeba histolytica/dispar *(*E. histolytica/dispar*) were not as prevalent (Table 1). The prevalence of helminth infections was higher in children from Oaxaca (53%) than in those from Sinaloa (33%) (p < 0.0001) (Table 2). The prevalence of protozoan infections in Sinaloa was the same as in Oaxaca (p = 0.39). A high prevalence of *E. coli *and *E. nana *was found in both groups. Prevalence of infection was not different by gender (p > 0.05)

Eggs counts of helminth infections (epg) was carried out on 116 (22.8%) schoolchildren (64 and 52 from Sinaloa and Oaxaca respectively) (Table 3). The arithmetic means of epg for *H. nana*, *A. lumbricoides*, and *T. trichiura*, were 9.5 (1.5–17.4), 25.3 (7.1–44.3) and 52 (33–71). The arithmetic means of epg by state for *H. nana*, *A. lumbricoides *and *T. trichiura *were 8, 38, and 54 respectively in Sinaloa and 10, 7, 35 respectively in Oaxaca (Table 3).

ANCOVA analysis showed higher logarithms of the egg counts for *A. lumbricoides *and *T. trichiura *in the schoolchildren of Sinaloa than in Oaxaca (0.44 vs 0.17 and 0.99 vs 0.41 respectively) (p < 0.01). No difference was found in the intensities of *H. nana *between both states (p > 0.05).

No difference was found in the education level and proportion of employed parents between the groups from Oaxaca and Sinaloa (Table 4). Block and/or cement and concrete were the most commonly used materials for households in Sinaloa, and metal/laminate most commonly used for roofs in Oaxaca. Defecation in open areas was a more frequent practice for children from Oaxaca (28%) than for children in Sinaloa (8%), and latrine use was more common in Sinaloa. Most of the interviewed parents in Oaxaca said they used boiled or chlorinated drinking water, whereas 53% of parents in Sinaloa reported using untreated water (Table 4).

Children from lower-income families were more likely to have intestinal parasitic infections (OR = 6.0, Table 5) compared with those from higher-income families. Maternal unemployment and low education level represented a higher risk of infection for children (OR = 4.5 and OR = 3.3 respectively, Table 5). Children who defecated in open areas were more likely to be infected than children who used pit toilets and latrines in both regions (OR = 2.4, Table 5). Stepwise logistic regression model was used to find the good subset of statistically significant predictors of infection used in the Table 5 (Table 6). Paternal employment and education, type of used materials for walls and roofs in the households and untreated drinking water did not appear in the last step model (Table 6).

## Discussion

Our study population provided data with 95% of confidence (expecting 50% of prevalence and 40% of non- participation) and was representative to populations with similar socio-economic status in the studied communities.

In this study, we looked at the prevalence of parasitic infections in schoolchildren in rural Mexican communities, and asked whether a number of socio-economic and environmental factors were related to the prevalence and intensity of infections. The intensities of *A. lumbricoides *and *T. trichiura *were statistically higher in the schoolchildren of Sinaloa than in those of Oaxaca. However, the prevalence of helminths infections was higher in the children of Oaxaca. It is known that environmental conditions are more conducive to development and transmission of helminths infections in Oaxaca, but the higher intensities found in Sinaloa may be associated not only to the environment conditions but also to re-infection rates, predisposition, or difference in the efficacy of the national albendazole administration.

Few epidemiological studies have been carried out in Sinaloa and Oaxaca, and those studies did not analyze the data according to demographic characteristics and socio-economic conditions. The present study revealed that the prevalences of *G. duodenalis, E. coli, E. nana, I. butschlii, T. trichuria, A. lumbricoides, E. vermicularis*, *E. histolytica *and *H. nana *in schoolchildren were similar to those found by Diaz *et al*. in 1987 and 1994 in the general population in rural and suburban communities in Sinaloa [16]. The low prevalence of *A. lumbricoides *in children from Oaxaca was probably a result of the albendazole given by the national campaign. The activity of a single dose of albendazole might be limited against protozoan parasites [17], but can improve cure rates and reduce egg counts of helminths such *A. lumbricoides*, hookworms, and *T. trichiura *[18].

We found that the prevalences of *G. duodenalis, T. trichiura, E. coli, E. nana*, and *I. butschlii *were higher than those reported in the general population of Oaxaca by Navarrete *et al*. in 1993 [19]. It is should be highlighted that protozoan infections, particularly those by *G. duodenalis *are an important cause of infection in these children.

No difference was found in the prevalence of infection between males and females in this study. Some studies have found a higher prevalence of particular parasites in females than in males, irrespective of age, such as in Papua New Guinea and in the Philippines where prevalence differences can be attributed to the different occupational activities of males and females [20,21]. A higher family income was related to a lower prevalence of parasitism in the children in our study. Studies in Iran [22,23]showed that the better the economic score of the family, the lower the prevalence of parasitic infection.

In this study, we asked whether toilet facilities and access to drinking water had an effect on rates of parasitic infection. Defecation in open areas was found to be associated with a higher risk of infection. This is in agreement with many earlier studies. In Panama, Holland et al. (1988) found that pit toilets or latrines reduced the risk of parasitic infection [24]. Prado et al. (2003) found that in Salvador, Brazil, a lack of a sewer in the house was significantly associated with *G. duodenalis *infection in 694 children aged from 2 to 45 months [25]. Properly functioning toilets installed inside the house reduced helminth infections in 319 individuals from Kuala Lumpur in Malaysia [26]. Proper hand washing and clean drinking water can reduce the transmission of helminth infections [27], and the type of drinking water (treated vs untreated) was significantly associated with intestinal parasitic infections in 551 individuals in Belize [28]. In our study safe drinking water and hand washing were not associated with infection. The latter might be explained by the inconsistency between what the parents said and what they really did in the household.

Prakash et al. (1980) [29] and Holland et al. (1988) [24] have highlighted that overcrowded conditions and particular types of house materials are associated with a high prevalence of parasitic infection. In this study, these factors were found not to be associated with infection. Most likely, the intra-family transmission of parasites is reduced by the national administration of albendazole, so that overcrowding does not have much impact on infection prevalence. Maternal, but not paternal, education levels were inversely correlated with the risk of infection in children in this study. This finding is in agreements with findings in studies in other developing nations. For example, Curtale et al., 1998 [10] found that the knowledge, perception, and behaviour of mothers were instrumental in designing and implementing an effective community-based intestinal helminth control program in Egypt. Wamani et al., 2004 [9] found that the mother's education was the best predictor of health and nutrition inequalities among children in rural Uganda. Finally, Nematian et al., 2004 [22] showed that the better the educational level of the mothers, the lower the parasitic infection rate in children in Iran. It is noteworthy that 55% and 45% of the fathers in the families from Sinaloa and Oaxaca respectively were absent from their homes for 6 months or more a year because of their employment. Maternal unemployment was a risk for infection in this study which is similar to data reported by Tucker in 1986 [30]. It would be expected that the more time mothers spend in the household, the better children's health. Sixty nine percent the mother in this study left children with grandmothers which could be related to a lower risk of parasitism.

There were no significant associations between the prevalence of protozoa or helminths with socio-economic factors which was probably due to the effect of dividing the sample into these categories. The same probably holds true for house materials, crowding index and type of drinking water.

In Egypt, there is increasing awareness in some communities about the importance of nutrition and hygiene education, especially for girls and mothers [10]. A community-based mothers' and infants' centre has been proposed to provide nutritional education and counselling for mothers from low income areas in Kota Emessu [31]. We propose that Mexican institutions should similarly promote health education to mothers in order to prevent and treat intestinal parasitic infections at home. Program managers and policy makers, as well as those running the Mexican national de-worming campaign, can more effectively target families with children at higher risk by targeting households in which the mother's educational levels are low. Such efforts will result in a direct and positive effect on the well-being of the young children.

## Conclusion

Intestinal parasites still pose an important public health problem in the Mexican states of Sinaloa (north-western Mexico) and Oaxaca (south-eastern Mexico). The prevalence of helminth infection was higher in the schoolchildren from Oaxaca than that in those from Sinaloa in spite of the national anti-helminth campaign. Lower income, defecation in open areas, unemployment and low education level of mothers were the significant factors associated with these infections. Our results can be used by appropriate authorities to target vulnerable families in Mexican rural communities, and should encourage the involvement of mothers in the activities of the national deworming campaign because intestinal parasitic infections will be difficult to control by drugs alone.

## Competing interests

The author(s) declare that they have no competing interests.

## Authors' contributions

LQ planned the research, performed the sampling, data input, and statistical analyses, and wrote the draft and final versions of the manuscript. MEV, contributed with financial assistance, lab space and discussions of the results and the manuscript. DWTC inspired the study, contributed with financial support, lab space and helped write the manuscript. SP contributed to discussions of the results and the manuscript. PH helped write the manuscript. GM helped with data input, preparing the tables and writing the manuscript. SPDC participated in the initial study design, sampling, and revising the manuscript. All authors read and approved the final manuscript.

## Pre-publication history

The pre-publication history for this paper can be accessed here:



## Figures and Tables

**Table 1:** The prevalence distributions of parasites of 507 rural schoolchildren 6–10 years from 12 rural communities of two Mexican states. 1997–2000.

Infection	Prevalence % (n)^f^	95%CI^a^
Polyparasitism^b^	57 (289)	53–61
Helminth Infection^c^	53 (269)	48–57
Protozoan Infection^d^	63 (319)	59–67
Pathogenic sp.		
*A. lumbricoides*	9 (46)	7–11
*T. trichiura*	16 (81)	13–19
*G. duodenalis*	24 (122)	20–28
*E. histolytica/dispar*	5 (25)	3–7
*H. nana*	23 (117)	19–27
Non-pathogenic sp.		
*E. coli*	46 (233)	42–50
*E. nana*	38 (193)	34–42

^a ^Confidence interval; ^b ^Infection with two or more pathogenic and/or non-pathogenic parasites; ^c ^Infection with helminths only; ^d ^Infection with protozoa only; ^f ^Number of children.

**Table 2:** Comparison of the prevalence of intestinal parasitic infections in 507 rural schoolchildren aged 6–10 years between two Mexican states.

	Sinaloa (n = 356)		Oaxaca (n = 151)		
	Prevalence% (n)^f^	95%CI^a^	Prevalence % (n)^f^	95%CI^a^	p value^e^

Polyparasitism^b^	51 (182)	42–60	54 (82)	48–59	0.77
Helminth Infection^c^	33 (117)	25–41	53 (80)	47–59	<0.0001
Protozoan Infection^d^	61 (217)	53–69	70 (106)	65–75	0.39
Pathogenic sp.					
*A. lumbricoides*	7 (25)	3–11	3 (5)	1–5	0.18
*T. trichiura*	11 (39)	6–17	12 (18)	8–16	0.89
*G. duodenalis*	31 (110)	24–39	23 (35)	19–28	0.22
*E. histolytica/dispar*	7 (25)	4–13	2 (3)	1–3	0.06
*H. nana*	25 (89)	20–29	23 (35)	16–30	0.81
Non-pathogenic sp.					
*E. coli*	40 (142)	32–49	53 (27)	48–58	0.11
*E. nana*	35 (125)	28–43	42 (63)	37–47	0.39

^a ^Confidence interval; ^b ^Infection with two or more parasites; ^c ^Infection with helminths only; ^d ^Infection with protozoa only; ^e ^Chi square test; ^f ^Number of children.

**Table 3:** Intensity of helminth infections in eggs per gram of faeces in 116 schoolchildren by state.

	Sinaloa (n = 64)	Oaxaca (n = 52)
Parasite	Arithmetic mean (interval confidence 95%)	

*H. nana*	8 (1.4–15)	10 (1.5–20)
*A. lumbricoides*	38 (6.6–70)	7 (1–11)
*T. trichiura*	54 (19–88)	35 (11–58)

**Table 4:** Socio-economic characteristics of 507 rural schoolchildren aged 6–10 years from 12 Mexican communities, 1997–2000.

Characteristics	Sinaloa n = 356	Oaxaca n = 151	p value
Mother			
Unemployed (%)	65 (231)^e^	59 (89)^e^	0.59^d^
Employed (%)	35 (125)^e^	41 (62)^e^	0.44^d^
Primary education in years	5 (3.7)^a ^(18)^e^	4.5 (2.8)^a ^(7)^e^	0.79^b^
Father			
Unemployed (%)	9 (32)^e^	10 (15)^e^	0.88^d^
Employed (%)	91(324)^e^	90 (136)^e^	0.99^d^
Primary education in years	4.0 (3.5)^a ^(14)^e^	3.8 (3.2)^a ^(6)^e^	0.70^b^
Household conditions			
Walls			
Block/cement (%)	67 (239)^e^	31 (47)^e^	<0.0001^d^
Adobe (%)	17 (61)^e^	26 (39)^e^	
Bamboo/mud (%)	12 (43)^e^	24 (36)^e^	
Board laminate/wood (%)	4 (14)^e^	19 (29)^e^	
Roof			
Concrete (%)	80 (285)^e^	13 (20)^e^	<0.0001^d^
Metal laminate/wood (%)	16 (57)^e^	83 (296)^e^	
Board laminate/wood (%)	4 (14)^e^	4 (14)^e^	
Floor			
Bare-earth (%)	37 (132)^e^	47 (71)^e^	0.20^d^
Cement (%)	63 (224)^e^	53 (80)^e^	0.32^d^
Crowding index^c^	4 (14)^e^	6 (9)^e^	0.34^b^
Sanitation facilities			
Defecation in Open Area (%)	8 (29)^e^	28 (42)^e^	<0.0001^d^
Pit (%)	68 (242)^e^	63 (95)^e^	
Latrine (%)	24 (85)^e^	9 (14)^e^	
Drinking water			
Treated water (%)	47 (167)^e^	70 (106)^e^	<0.01^d^
Untreated water (%)	53 (189)^e^	30 (45)^e^	<0.01^d^
Family income	4 times minimum daily wage	1 minimum daily wage	<0.001^b^

^a ^Mean (standard deviation); ^b ^Student t-test; ^c ^Mean of number people per room; ^d ^Chi square test; ^e ^Frequency or number of individuals.

**Table 5:** Association of socio-economic variables with intestinal parasitic infections in 507 schoolchildren aged 6–10 years old from Sinaloa (n = 356) and Oaxaca (n = 151).

Socio-economic variables	n	OR*	95%CI*
Family monthly income			
>2 minimum daily wage^a^	237	1.0	
<2 minimum daily wage	270	6.0	1.6–22.6
Mother			
Laboral activity			
Employed^a^	187	1.0	
Unemployed	320	4.5	2.5–8.2
Primary education			
Complete^a^	190	1.0	
Incomplete	317	3.3	1.5–7.4
Defecation			
Pit/latrine^a^	436	1.0	
Open area	71	2.4	2.0–3.0
Floor			
Cement^a^	304	1.0	
Bare-earth	203	0.9	0.6–1.3
Gender			
Male^a^	274	1.0	
Female	233	0.7	0.5–1.0
Age	507	0.6	0.3–1.1

^a ^Reference;*Multinomial logistic regression.

**Table 6:** The socio-economic predictors of intestinal parasitic infections in 507 schoolchildren (6–10 years old) from 12 rural communities of two Mexican states.

Socio-economic variables	Coefficient (standard error)	p* value
Family monthly income	1.80 (0.66)	<0.001
Mother unemployed	1.5 (0.3)	<0.001
Mother with incomplete primary school	1.2 (0.4)	<0.001
Defecation in open area	0.90 (0.1)	<0.001
Bare earth floor	-0.1 (0.2)	<0.001
Crowding index	-0.30 (0.1)	<0.001
Gender	-0.4 (0.2)	<0.001
Age	-0.5 (0.3)	<0.01

*Multiple regression analysis (stepwise-backward elimination). Only significant regression coefficients (β) are shown. R^2 ^= 0.78
